# Fear of COVID-19 and PTSD: The Protective Function of Problem-Solving Appraisals in Mental Health

**DOI:** 10.3390/ijerph21020220

**Published:** 2024-02-13

**Authors:** Anita Padmanabhanunni, Tyrone Brian Pretorius

**Affiliations:** Department of Psychology, University of the Western Cape, Western Cape, Cape Town 7530, South Africa; tpretorius@uwc.ac.za

**Keywords:** fear of COVID-19, problem-solving appraisal, PTSD, anxiety, students

## Abstract

The COVID-19 pandemic was experienced by many people as a major traumatic event, and it contributed to high levels of fear, anxiety, and PTSD. Negative cognitive appraisals have been consistently implicated in the onset and maintenance of psychological distress, but there is far less research on the protective role of adaptive appraisals in mental health outcomes. The current study aimed to address this gap by investigating the role of problem-solving appraisals in the relationship between fear of COVID-19 and PTSD. Participants were students (*n* = 322) who completed the Fear of COVID-19 Scale, the Problem-Solving Inventory, the PTSD Checklist for DSM-5, and the five-item short version of the trait scale of the State–Trait Anxiety Inventory. Participants had a mean age of 26 years (±10.2; range 17–63). The results revealed that problem-solving appraisal mediated the effects of fear of COVID-19 on all the dimensions of PTSD. However, moderated mediation analysis demonstrated that the mediation effect was moderated by anxiety. In this regard, the indirect effects of fear of COVID-19 on PTSD were only significant for respondents with low anxiety levels. Our findings suggest that intervention efforts need to focus on identifying and actively targeting maladaptive appraisals of the problem-solving ability as well as addressing anxiety-related symptoms that may impede coping.

## 1. Introduction

The COVID-19 pandemic represents a global public health emergency. The outbreak led to governments around the world implementing a range of measures to curb the spread of infection, including national lockdowns, restrictions on movement, mandatory social distancing policies, and work-from-home mandates [1]. The current study was undertaken in South Africa, where pandemic-related restrictions were particularly stringent, affecting various aspects of daily life and exacerbating existing socio-economic challenges. These restrictions had a significant impact on the higher education sector and university students [2]. In response to the pandemic, universities swiftly transitioned to online platforms to continue educational activities. This shift involved a range of adaptations, including virtual classrooms, digital submission of assignments, and online examinations. While necessary, these changes introduced new stressors for students, such as digital divide issues, the adjustment to remote learning, and loss of direct social interactions, potentially exacerbating feelings of isolation and anxiety [3,4].

During the pandemic, the experiences of students regarding access to mental health care resources varied widely [5,6,7]. While physical restrictions limited traditional in-person engagement with social networks and health care providers, leading to reduced access for some, the crisis also catalyzed the expansion of online mental health services [8]. Many countries saw a surge in digital health initiatives, with experts and therapists offering online consultations and therapy sessions for the first time [8,9]. This shift compensated for the loss of face-to-face interactions and introduced new avenues for mental health support, making services more accessible to some student populations. Nevertheless, the effectiveness and accessibility of these online services varied, reflecting disparities in digital literacy, internet access, and personal preferences for types of mental health care engagement [5,6,7]. The characteristics of the outbreak, including unprecedented infection rates, high numbers of critically ill patients, and the occurrence of different variants of the disease, aggravated psychological distress [10]. In developing countries, challenges with the provision of personal protective equipment, an economic down-turn, inequalities in access to digital resources, job losses, and concerns about food security added to the mental health burden associated with the pandemic [2,11].

Fear has been identified as a central emotional response to the pandemic [12,13]. A Jordanian study reported a 52.7% prevalence rate for fear among the population, and this was related to concerns about the health implications of being infected and worries about the wellbeing of loved ones in the event of contagion [14]. A cross-sectional study in Bangladesh found a prevalence rate of 86.3% among employed adults, and these elevated levels of fear were attributed to the negative economic impact of the pandemic on the employment sector and concerns about job security [15]. A South African study reported higher levels of fear among a sample of school teachers compared to studies undertaken in other low-to-middle income countries [16]. A systematic review and meta-analysis of studies on psychological distress among student populations reported a prevalence rate of 33% for fear [17]. Difficulties engaging with emergency remote online learning, delayed academic progress, and prolonged lack of social engagement were some of the factors contributing to heightened fear among this population group.

Although fear can be adaptive when faced with a significant and potentially life-threatening stressor, excessive levels of fear can adversely impact on mental health and lead to heightened levels of anxiety following disease outbreaks [18,19]. Anxiety, particularly as a trait, predisposes individuals to perceive a range of situations as threatening, thereby amplifying the stress associated with the pandemic’s uncertainties [20]. Individuals with high trait anxiety are more likely to perceive situations as threatening, even when they might not be, and this heightened sensitivity can lead to increased stress and worry about the pandemic’s consequences [20]. Furthermore, trait anxiety can potentially influence cognitive processes involved in evaluating one’s ability to manage or cope with stressful situations. Individuals with high trait anxiety might be more prone to negative appraisals of their coping abilities, viewing challenges as insurmountable or beyond their control, which can further contribute to psychological distress [20].

Trait anxiety not only strains an individual’s mental health but can also lead to long-term consequences such as chronic anxiety disorders, depression, or PTSD, especially when combined with direct or indirect experiences of loss, illness, or significant life changes due to the pandemic [21,22]. Various studies have highlighted the prevalence of PTSD following disease outbreaks. For example, Gao and colleagues assessed for PTSD among SARS survivors and reported rates of PTSD ranging from 38.8% to 46.2% [23]. Kaputu-Kalala-Malu and colleagues found the prevalence of PTSD was at 24.3% among survivors of Ebola disease in Sierra Leone [24]. A systematic review and meta-analysis on the prevalence of PTSD following infectious disease pandemics, including COVID-19, reported a 26.2% and 27.2% pooled prevalence of PTSD among males and females, respectively [25]. Based on their meta-analysis of studies undertaken in a wide range of countries (e.g., Bolivia, China, Italy, Spain, France, and the United States), Cénat and colleagues reported a pooled prevalence rate of 21.94% and 13.29% for PTSD and psychological distress, respectively [26]. In their meta-analysis, Yunitri and colleagues found that the average PTSD prevalence across 24 countries, including France, Greece, Norway, and Italy, was 17.52% [27].

Several studies have also confirmed the prevalence of PTSD among university students during the COVID-19 pandemic [26,28]. PTSD is a complex psychiatric disorder characterized by intrusive memories of the traumatic event or its sequalae, alternations in cognition and mood, physiological hyper-arousal, and cognitive and behavioral avoidance of reminders of trauma [29]. If untreated, the condition can severely impact on interpersonal, occupational, and educational functioning. Pandemic-related factors that have been found to increase fear, anxiety, and vulnerability to PTSD include appraisals of increased risk of contagion to oneself and loved ones, having family members or significant others who have been infected, quarantine and social isolation due to infection, and economic loss that impacts on livelihood [25,30].

This study is grounded in the Cognitive Transactional Model (CTM) of stress and coping, which proposes that cognitive appraisals are central in determining emotional responses to stressors and influencing outcomes [31]. Appraisals entail an evaluation of the stressful event or situation as relevant to the individual’s goals and determining how to negotiate the stressor so as to improve chances of coping effectively. This approach involves continuous interactions or ‘transactions’ between the individual and environment [31]. Different types of cognitive appraisals have been found to be differentially associated with emotional and behavioral responses [32,33]. For example, Padmanabhanunni and Wiid [33] reported that fortigenic or adaptive appraisals of self, family, and significant others were associated with reduced levels of PTSD symptoms, including decreased intrusive re-experiencing and avoidance behaviors. Li and colleagues [34] reported that appraisals of controllability were influential in predicting levels of distress and behavioral responses towards the COVID-19 outbreak among the Chinese population. A Turkish study [35] found that appraisals of self-efficacy were related to mental health, while Prasetyo and colleagues [36] reported that perceptions of the effectiveness of COVID-19 prevention measures and appraisals of vulnerability influenced mental health outcomes among Filipinos. Using the CTM framework, the current study aimed to advance research on the role of cognitive appraisals in mental health outcomes, by examining the potential mediating role of problem-solving appraisal in the relationship between fear of COVID-19 and PTSD.

Problem-solving appraisal refers to the individual’s perception of their problem-solving abilities rather than their actual problem-solving skills [37]. The majority of studies on the role of problem solving in mental health have focused on problem-solving ability [38,39]. However, appraisal of problem-solving ability influences the choice of coping responses and actual problem-solving skills. Individuals who appraise themselves as inadequate in problem solving may be hesitant and unmotivated to engage in solving problems. Hepper and colleagues [40], in a review and synthesis of the literature focusing on the relationship between problem-solving appraisal and psychological adjustment, concluded that there was a strong association between problem-solving appraisal and a wide range of indices of psychological wellbeing. Subsequent studies have confirmed this finding [41,42]. We hypothesized that problem-solving appraisal will mediate the association between fear of COVID-19 and the dimensions of PTSD as well as the association between anxiety and PTSD.

## 2. Materials and Methods

### 2.1. Research Context

The current study was undertaken at an historically disadvantaged university in South Africa. Historically disadvantaged institutions or HDIs were established by the apartheid government for black South Africans and were significantly under-resourced [30]. Although there has been significant transformation in the educational sector in the country since democratization, the majority of students at HDIs are from working class backgrounds [20]. This is due to historical factors, as well as the comparatively lower fees charged by these institutions. A significant proportion of students at HDIs reside in disadvantaged community contexts characterized by high levels of gang violence, poverty, and substance abuse [20]. This increases their vulnerability to PTSD, and studies (e.g., [31]) have reported higher incidences of traumatic stress reactions among students at HDIs.

### 2.2. Participants and Procedure

Participants were students (*n* = 322) at a single university in the Western Cape Province, South Africa. They were randomly sampled through the Registrar’s office. We used Google Forms to create a web-based survey consisting of all the instruments. The medium of instruction at the university is English, and the survey was administered in the English language. The Registrar’s office randomly selected the email addresses of 1500 students and sent the link to them. Our sample constitutes a response rate of 21.5%. Similar response rates have been reported in other studies [22,32]. There were no missing data. The majority of the sample resided in an urban area (87.3%) and were women (77%). The mean age of the sample was 26 years (±10.2; range 17–63).

### 2.3. Instruments

Participants were administered several psychometric instruments to assess their psychological responses to the pandemic and related stressors. These included the Fear of COVID-19 Scale (FCV-19S: [43]), the Problem-Solving Inventory (PSI: [37]), the PTSD Checklist for DSM-5 (PCL-5: [44]), and the 5-item short version of the trait scale of the State–Trait Anxiety Inventory (Stai-T5: [45]). In addition, participants also completed a brief demographic questionnaire.

The FCV-19S assesses fear reactions in relation to the pandemic and consists of seven items, measured on a five-point scale ranging from 1 (strongly disagree) to 5 (strongly agree), allowing for a cumulative score that reflects the level of fear. An example of a scale item includes, “I am most afraid of coronavirus-19”. Ahorsu and colleagues [43] reported satisfactory reliability coefficients (0.82) in the scale development study and the relationship between fear of COVID-19 and anxiety and depression, as well as perceived vulnerability to disease, provided evidence for the validity of the scale. In South Africa, the FCV-19S demonstrated sound psychometric properties in both a student and teacher sample [16,46].

The PSI measures an individual’s perception of their problem-solving skills rather than their actual problem-solving skills. It consists of 32 items that are scored on a 6-point scale that ranges from 1 (strongly agree) to 6 (strongly disagree). The PSI is scored in such a way that higher scores are reflective of the respondents’ appraisal of themselves as being ineffective problem solvers. Only total scores for the PSI were used in this study. An example of an item includes, “I make snap judgments and later regret them”. In the original study that reported on the development of the PSI, Heppner and Petersen [47] reported a reliability estimate of 0.90, and correlations with students’ rating of their problem-solving skills and their level of satisfaction with their problem-solving skills served as evidence for validity. The PSI has been used in a range of studies in South Africa [48,49], and reliability estimates typically ranged between 0.83 and 0.94.

The PCL-5 assesses the presence and severity of PTSD symptoms. It is a 20-item scale that has a 5-point response format ranging from 0 (not at all) to 4 (extremely). The items of the PCL-5 correspond to the DSM-5 criteria for PTSD. The PCL-5 has four subscales: re-experiencing (spontaneous and intrusive memories of the traumatic event, 5 items); avoidance (distressing memories, thoughts, feelings, or external reminders of the event, 2 items); negative alterations in mood and cognition (a range of feelings including a persistent and distorted sense of blame of self or others, being estranged from others, or significantly reduced interest in activities, 7 items); and hyper-arousal (aggressive, reckless, or self-destructive behavior, sleep disturbances, hyper-vigilance or related problems, 6 items). Higher scores indicate heightened levels of PTSD symptomology. An example of an item includes, “In the past month, how much were you bothered by repeated, disturbing, and unwanted memories of the stressful experience?” The authors of the PCL-5 reported satisfactory estimates of internal consistency of 0.94 and 0.95 in two separate studies. In addition, the strong correlations between the PCL-5 and other self-report measures of PTSD provided evidence for convergent validity, while moderate correlations with related constructs, such as depression, served as evidence for discriminant validity. A South African study reported a Cronbach’s alpha of 0.93 for the PCL-5 when used with students [33].

The STAI-T5 is 5-item version of the original 20-item trait scale of the State–Trait Anxiety Inventory [50]. It assesses trait anxiety and consists of 5 items scored on a 4-point scale ranging from 1 (not at all) to 4 (very much so). Higher scores indicate higher levels of anxiety. An example of an item from the scale includes, “Difficulties are piling up”. Zsido and colleagues reported a reliability coefficient of 0.86 for the reduced-item version that was comparable to that of the original 20-item version (0.88) [45]. Validity was demonstrated by the significant correlations between the STAI-T5 and depression, life satisfaction, and self-esteem. We could not find any studies in South Africa that has used the short version of the STAI-T.

### 2.4. Ethics

The current study was conducted in accordance with the guidelines of the Declaration of Helsinki. Ethical approval for the study was provided by the Humanities and Social Sciences Ethics Committee of the University of the Western Cape (ethics reference number: HS22/2/9, February 2022). Participation was voluntary and anonymous, and participants had to provide informed consent on the landing page of the web-based survey. No incentives were offered for participation in the study.

### 2.5. Data Analysis

All items of the questionnaire were marked as compulsory, and participants could not proceed with the electronic questionnaire if an item was not completed. Thus, there were no missing data. All statistical analyses were conducted with IBM SPSS Statistics version 26 for Windows (IBM Corp., Armonk, NY, USA). This included indices of skewness and kurtosis, descriptive statistics (means and SD), intercorrelations between variables (Pearson r), and estimates of reliability (Cronbach’s alpha). With respect to the distribution of scores, skewness values between −2 and +2 and excess kurtosis values between −7and +7 would indicate that the data is approximately normally distributed [51]. The PROCESS macro in SPSS [42] was used to examine the mediating role of problem-solving appraisal in the relationship between fear of COVID-19 and PTSD (PROCESS Model 4), and to examine whether anxiety moderated this mediational relationship through moderated mediation analysis (PROCESS Model 7: see Figure 2). In the moderated mediation analysis, the variables that were used to create the interaction term (fear of COVID-19 and anxiety) were mean-centered. The nature of the moderating effect was plotted for three levels of anxiety, namely, −1SD, mean, +1SD on the moderator variable. The plots of these three groups were generated using the visualization code provided by PROCESS. The significance of the index of moderated mediation, provided by PROCESS, identify in which instances the indirect effect of fear of COVID-19 on PTSD was moderated by anxiety. Where the index of moderated mediation was significant, PROCESS provides a conditional indirect effect for each of the three levels of anxiety.

## 3. Results

The descriptive statistics, intercorrelations, and reliability of study variables are reported in Table 1. The indices of skewness and kurtosis indicated that the scores for all instruments were approximately normally distributed, as these indices were in the recommended range. All the scales demonstrated satisfactory reliability, ranging from 0.82 to 0.89.

Table 1 also reflects that fear of COVID-19 was positively associated with problem-solving appraisal (r = 0.12, *p =* 0.04) and all the dimensions of PTSD (re-experiencing: r = 0.28, *p* < 0.001; avoidance: r = 0.26, *p* < 0.001; negative alterations in mood and cognition: r = 0.26, *p* < 0.001; hyper-arousal: r = 0.26, *p* < 0.001), as well as anxiety (r = 0.20, *p* < 0.001). This would indicate that higher levels of fear of COVID-19 are associated with self-perceptions of ineffective problem solving (higher scores on the PSI are reflective of perceptions of ineffective problem solving), higher levels of PTSD, and higher levels of anxiety. Problem-solving appraisal was positively related to all dimensions of PTSD (re-experiencing: r = 0.35, *p* < 0.001; avoidance: r = 0.28, *p* < 0.001; negative alterations in mood and cognition: r = 0.51, *p* < 0.001; hyper-arousal: r = 0.43, *p* < 0.001) and anxiety (r = 0.48, *p* < 0.001). Thus, perceptions of ineffective problem solving are associated with higher levels of PTSD as well as anxiety.

The mean scores for re-experiencing, avoidance, negative alterations in mood and cognition, hyper-arousal, and anxiety, expressed in terms of the 5-point scale of the PCL-5, were 1.9, 2.15, 1.93, and 1.87, respectively. In terms of the prevalence of PTSD, a cut-off score of 31 was used, as suggested by Ashbaugh and colleagues, and it was found that 62.1% would meet a provisional diagnosis of PTSD [52].

The direct effects of the fear of COVID-19 and problem-solving appraisal on the indices of PTSD are presented in Table 2. All of the effects were significant and confirm the associations obtained with the zero-order correlations.

The mediation analysis confirmed a significant mediating role for problem-solving appraisal in the relationship between fear of COVID-19 and all the dimensions of PTSD, namely, re-experiencing (β = 0.03, 95% CI [0.002, 0.065]), avoidance (β = 0.01, 95% CI [0.000, 0.026]), negative alterations in mood and cognition (β = 0.06, 95% CI [0.002, 0.111]), and hyper-arousal (β = 0.04, 95% CI [0.001, 0.087]).

**Figure 1:**
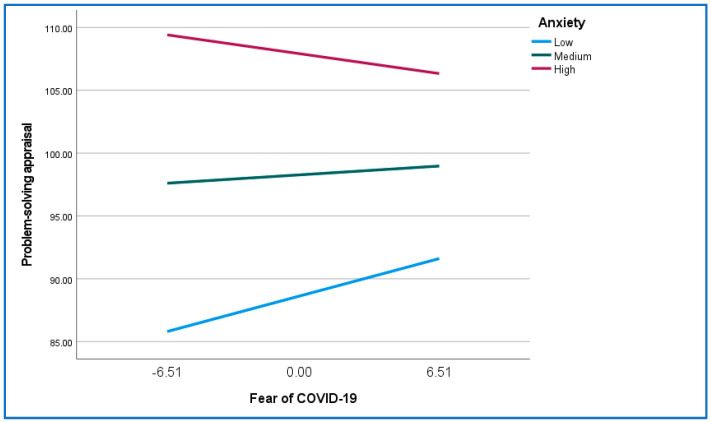
The relationship between fear of COVID-19 and problem-solving appraisal for high, medium, and low levels of anxiety.

The results of the moderated mediation analysis found a significant interaction effect (fear of COVID-19 X anxiety: β = −0.08, *p* = 0.02)), indicating that anxiety moderated the relationship between fear of COVID-19 and problem-solving appraisal. The nature of the moderation effect is demonstrated in Figure 1.

For respondents with high levels of anxiety, the regression line is in the opposite direction of the regression lines for low or medium levels of anxiety. This would indicate that for participants with high anxiety, increased levels of fear of COVID-19 are associated with a decrease in problem-solving appraisal (i.e., perceptions of effective problem-solving skills). Simple slope tests indicated that, at low levels of anxiety, there was a significant positive association between fear of COVID-19 and problem-solving appraisal (β = 0.46, *p* = 0.04), while at medium (β = 0.10, *p* = 0.50) and high (β = −0.24, *p* = 0.24) levels of anxiety, the association between fear of COVID-19 and problem-solving was not significant.

**Figure 2:**
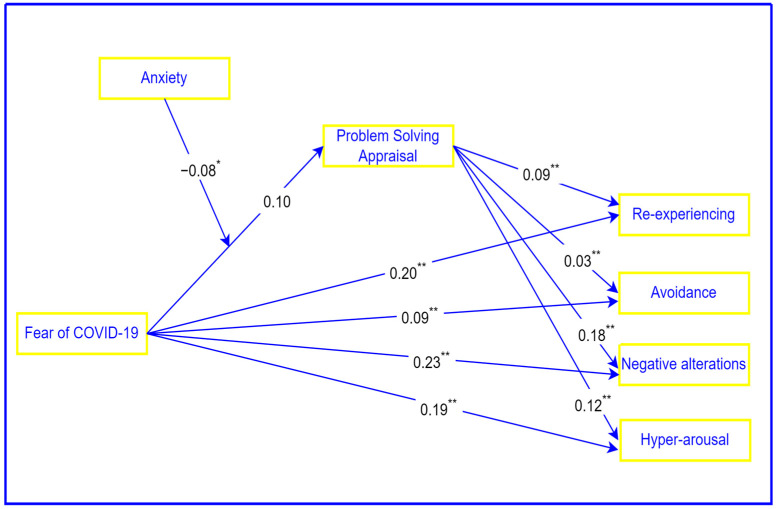
Mediating role of problem-solving appraisal in the relationship between fear of COVID-19 and PTSD, and the moderating role of anxiety. ** *p* < 0.001, * *p* < 0.05.

The moderated mediation model is shown in Figure 2.

The indices of moderated mediation for all the dimensions of PTSD are presented in Table 3. All the indices were significant, which indicates that the indirect effect of fear of COVID-19 on the dimensions of PTSD was moderated by anxiety.

The conditional indirect effects of fear of COVID-19 on the dimensions of PTSD for different levels of anxiety are presented in Table 4. For all the dimensions of PTSD, the indirect effects of fear of COVID-19 were significant for respondents with low levels of anxiety but not for those with medium or high levels of anxiety. This confirms that the mediating role of problem-solving appraisal was moderated by anxiety. Thus, while a significant mediation effect was found for problem-solving appraisal for all the indices of PTSD in the mediation analysis, the effect was only significant for respondents with low levels of anxiety in the moderated mediation analysis.

## 4. Discussion

The COVID-19 pandemic has been associated with a range of adverse mental health outcomes [3,53]. Fear has been identified as a dominant emotional response to the outbreak of the disease, and elevated levels of fear have been associated with adverse mental health outcomes, including anxiety, depression, and PTSD [17,54]. Existing research has highlighted the critical role of cognitive appraisals in emotional regulation and psychological distress. The bulk of this research has focused on the role of negative appraisals, with less focus on the protective function of adaptive appraisals [55,56]. These types of appraisals can serve a protective function and mitigate the development of adverse mental health outcomes. The current study aimed to extend research in this area by examining the potential mediating role of problem-solving appraisal in the relationship between fear of COVID-19 and PTSD. There were several important findings.

First, the study confirmed that fear of COVID-19 was positively associated with all dimensions of PTSD. It is well established that fear is a dominant emotional reaction to traumatic events, and theoretical models of PTSD have proposed that impaired processing of the traumatic event in memory and the acquisition of fear through classical conditioning processes underlie the persistence of the disorder [57,58,59]. In the context of the COVID-19 pandemic, fear may be related to uncertainty about the course of the outbreak, the rapid transmissibility of the virus, the high mortality rates, and concerns about the health consequences of infection for oneself and family members. Those infected with the virus, or who suspect that they may have been infected, have been found to experience more intense levels of fear and PTSD [60]. Furthermore, individuals with a prior history of exposure to trauma are more vulnerable to experiencing fear and developing PTSD after exposure to subsequent potentially traumatic situations [23]. The current study consisted of students from a South African HDI, and prior research has confirmed increased levels of trauma exposure among this group because they reside in high-risk environments characterized by community violence, unemployment, poverty, and substance abuse [61]. Furthermore, limited access to personal protective equipment and health care resources in such settings may have aggravated their fears of COVID-19 and increased their risk of PTSD. It is possible that prior exposure to trauma may have enhanced their vulnerability to fear and PTSD in the context of COVID-19.

Second, the study found that stronger cognitive appraisals of being an effective problem solver were associated with lower levels of both fear of COVID-19 and PTSD. This finding underscores the protective role of adaptive appraisals in promoting emotional regulation and coping. Individuals who appraise themselves as ineffective problem solvers are more likely to engage in maladaptive emotion regulation strategies such as ruminative thinking (i.e., passive focusing on stressors and their causes), thought suppression, and behavioral avoidance [62]. These types of strategies have been implicated in the maintenance of fear and PTSD because they prevent individuals from processing traumatic events [57]. In contrast, the use of strategies such as the positive re-appraisal of stressful situations has been found to promote effective coping and enhance wellbeing [63].

Third, the study found that anxiety moderated the relationship between fear of COVID-19 and problem-solving appraisal. This suggests that for individuals with high anxiety, increased levels of fear of COVID-19 were associated with a decrease in problem-solving appraisal (i.e., perceptions of effective problem-solving skills). In the absence of the moderator (anxiety), problem-solving appraisal mediated the relationship between fear of COVID-19 and the dimensions of PTSD. Furthermore, when the moderator (anxiety) was introduced, the indirect effects of fear of COVID-19 on PTSD were only significant for those with low levels of anxiety. Cognitive models of emotional regulation have proposed that strong negative emotional experiences can interfere with executive functions such as cognitive processing, appraisal, and planning [57,58]. When applied to our findings, it is probable that high levels of anxiety may have negatively impacted cognitive appraisals of self and problem-solving ability.

The findings that anxiety moderated the relationship between fear of COVID-19 and problem-solving appraisal also aligns with personality theories suggesting that individuals with high trait anxiety are more likely to experience diminished perceptions of their problem-solving abilities in the face of stress [64]. This is particularly relevant in the context of the COVID-19 pandemic, where the unprecedented nature of the outbreak and drastic changes to the educational sector have presented significant stressors. Students with high trait anxiety may perceive these challenges as more threatening, impacting their cognitive appraisals and, consequently, their ability to engage in effective problem solving. This relationship underscores the importance of considering personality-based explanations in understanding how students cope with pandemic-related stress, highlighting the need for interventions that are tailored to individual personality profiles to enhance resilience and coping mechanisms in the face of ongoing and future stressors [64].

The findings of the study have potential implications for interventions aimed at promoting adaptive coping among university students. Existing studies have demonstrated that problem-solving ability can be cultivated through training and practice, signifying that this protective factor can be a target of intervention efforts [65,66]. Evidence-based treatments for psychological disorders, such as cognitive behavioral approaches, emphasize increasing emotional regulation strategies and effective coping through positive reappraisals of potentially stressful situations [66]. Based on our findings, an important component of such an approach entails identifying and actively targeting maladaptive appraisals of problem-solving ability. This has the potential to generate more positive emotions, enhance confidence, and broaden an individual’s range of coping responses. Research-based support for the role of problem-solving training has been growing in recent years, and a central component of this approach entails focusing on enhancing appraisals of problem-solving ability [67]. The finding that anxiety moderated the relation between fear of COVID-19 and problem-solving appraisal implies that assessing for anxiety needs to be a part of intervention efforts.

The findings of the study need to be viewed in the context of several limitations. First, the study used a predominantly homogenous sample of students from the same institution, and additional research using a more diverse sample is warranted. Second, the study design is cross-sectional, limiting the extent to which causal inferences can be made. Longitudinal studies that assess student trajectories over time are needed to better understand the interplay of protective factors and mental health outcomes. Third, the study used an electronically distributed self-report instrument, and it is probable that students with access to information technology and interest in the topic may have been more likely to complete it. This may have contributed to selection bias and social desirability bias. Finally, the study did not assess trauma exposure or adverse events, and, while our use of the PCL-5 is commensurate with other studies involving university students [55], interpretations of the findings of the PCL as indicators of PTSD need to be made with caution. Furthermore, it is possible that factors apart from the COVID-19 pandemic may have contributed to anxiety and traumatic stress symptoms. Future studies would need to assess the influence of factors not measured in this study, including trauma exposure. 

## 5. Conclusions

The current study extends research on the role of adaptive cognitive appraisals in promoting coping among university students during the COVID-19 outbreak. The measurement of problem-solving appraisal in the context of the pandemic represents an important contribution to the literature in this area. Our hypothesis regarding the mediating effect of problem-solving appraisal on dimensions of PTSD was supported. However, this effect was moderated by anxiety. Furthermore, the indirect effects of fear of COVID-19 on PTSD were only significant for respondents with low anxiety levels. Interventions targeting anxiety may contribute to promoting student mental health.

## Figures and Tables

**Table 1:** Descriptive statistics for, reliabilities of, and intercorrelations between study variables.

Variables and Indices	1	2	3	4	5	6	7
1. Fear of COVID-19							
2. Problem-solving appraisal	0.12 *						
3. Re-experiencing	0.28 **	0.35 **					
4. Avoidance	0.26 **	0.28 **	0.66 **				
5. Negative alterations	0.26 **	0.51 **	0.12 **	−0.59 **			
6. Hyper-arousal	00.26 **	0.43 **	0.67 **	−0.54 **	0.80 **		
7. Anxiety	0.20 **	0.48 **	−0.54 **	0.48 **	0.65 **	0.59 **	
Mean	17.4	97.8	9.5	4.3	13.5	11.2	12.4
*SD*	6.5	20.3	5.5	2.6	7.5	6.0	4.1
Skewness	0.35	−0.08	0.04	−0.21	0.02	0.02	0.03
Kurtosis	−0.44	−0.05	−0.95	−1.18	−1.04	−0.91	−0.88
Alpha	0.87	0.89	0.89	0.89	0.88	0.82	0.88

** *p* < 0.001, * *p* < 0.05.

**Table 2:** Direct effects of fear of COVID-19 and problem-solving appraisal on PTSD.

Effect	Beta	SE	*p*	95% CI	
				LL	UL
Fear of COVID-19—re-experiencing	0.20	0.04	0.000	0.12	0.29
Fear of COVID-19—avoidance	0.09	0.02	0.000	0.05	0.13
Fear of COVID-19—negative alterations	0.23	0.05	0.000	0.13	0.34
Fear of COVID-19—hyper-arousal	0.19	0.05	0.000	0.11	0.28
Problem-solving appraisal—re-experiencing	0.09	0.01	0.000	0.06	0.11
Problem-solving appraisal—avoidance	0.03	0.01	0.000	0.02	0.05
Problem-solving appraisal—negative alterations	0.18	0.02	0.000	0.15	0.21
Problem-solving appraisal—hyper-arousal	0.12	0.01	0.000	0.09	0.15

**Table 3:** Indices of moderated mediation (anxiety).

Outcome Variable	Index	Bootstrapped SE	Bootstrapped 95% CI	
			LL	UL
Re-experiencing	−0.007	0.003	−0.013	−0.002
Avoidance	−0.003	0.001	−0.005	−0.001
Negative alterations	−0.015	0.006	−0.026	−0.003
Hyper-arousal	−0.010	0.004	−0.018	−0.002

**Table 4:** Conditional indirect effects of fear of COVID-19 on PTSD at different levels of anxiety.

Anxiety	Effect	SE	95% Confidence Interval	
			LL	UL
Re-experiencing				
Low anxiety	0.038	0.02	0.001	0.082
Medium anxiety	0.009	0.01	−0.012	0.040
High anxiety	−0.020	0.02	−0.053	0.014
Avoidance				
Low anxiety	0.014	0.008	0.000	0.032
Medium anxiety	0.003	0.006	−0.007	0.015
High anxiety	−0.008	0.007	−0.021	0.005
Negative alterations				
Low anxiety	0.080	0.041	0.003	0.159
Medium anxiety	0.019	0.029	−0.037	0.077
High anxiety	−0.042	0.035	−0.111	0.029
Hyper-arousal				
Low anxiety	0.054	0.028	−0.001	0.112
Medium anxiety	0.013	0.020	−0.025	0.054
High anxiety	−0.028	0.023	−0.075	0.019

## Data Availability

The raw data supporting the conclusions of this article will be made available by the authors, without undue reservation.
